# Primary Adrenal Angiosarcoma and its Management: A Case Report

**DOI:** 10.7759/cureus.48762

**Published:** 2023-11-13

**Authors:** Omar M Alsarraj, Awadh Alqahtani, Mohammed Alswayyed, Mohammad Almayouf, Srikar Billa

**Affiliations:** 1 Department of Surgery, Dr. Sulaiman Al-Habib Hospital, Al Takhasussi, SAU; 2 Department of Surgery, King Saud University, Riyadh, SAU; 3 Department of Pathology and Laboratory Medicine, King Saud University, Riyadh, SAU; 4 Department of Surgery, Prince Sattam bin Abdulaziz University, Alkharj, SAU

**Keywords:** surgery, malignancy, adrenalectomy, angiosarcoma, primary adrenal angiosarcoma

## Abstract

Primary adrenal angiosarcoma is a very rare malignancy. This is a case report of a 54-year-old female, who presented with right-sided abdominal pain. Magnetic resonance imaging of the abdomen and pelvis showed a right adrenal mass with a maximum dimension of 5.7 cm. The patient went for a laparoscopic right adrenalectomy. The postoperative period was uneventful, and she was discharged on postoperative day 2. The patient was free from complaints at outpatient follow-up visits. Pathology confirmed the diagnosis of adrenal angiosarcoma and the metastasis workup was negative. A multidisciplinary approach through the expertise of medical oncology, surgical oncology, and histopathology is essential for the diagnosis and management of such rare diseases.

## Introduction

Angiosarcoma is a very rare high-grade neoplasm that originates from the endothelium of blood vessels and lymphatic drains in addition to soft tissue, which commonly occurs in the breast, skin, spleen, bone, and liver. This type of malignancy accounts for less than 1% of all soft tissue sarcomas [1-3]. The five-year survival rate is 24-31% [4]. Primary angiosarcoma of the adrenal gland is highly rare, and just 51 cases of primary adrenal angiosarcomas have been reported in the medical literature from 1988 to 2021 [5]. This report describes the management of a patient who presented with a right adrenal mass, which was found to be a primary angiosarcoma.

## Case presentation

A 54-year-old female known to have diabetes mellitus, hypothyroidism, and ischemic heart disease on antiplatelet medication (clopidogrel 75mg, once daily) presented to our clinic with right-sided abdominal pain, which started one and a half months ago. It was mild pain and well responding to over-the-counter painkillers, and then became more severe over the past two weeks, with a history of multiple emergency department visits to the nearest dispensary. Due to its limited facilities, the patient was managed only by intravenous painkillers, which provided mild relief for her pain. No history of nausea, vomiting, fever, change in bowel habits, or urine characteristics. She denied any history of recent weight loss, anorexia, or fatigue. On examination, she looked stable, awake, and oriented but distressed and in severe pain. The abdomen was soft and lax, with mild epigastric tenderness, right side fullness/distension, and severe positive tenderness upon palpating the right costo-vertebral angle. 

**Figure 1:**
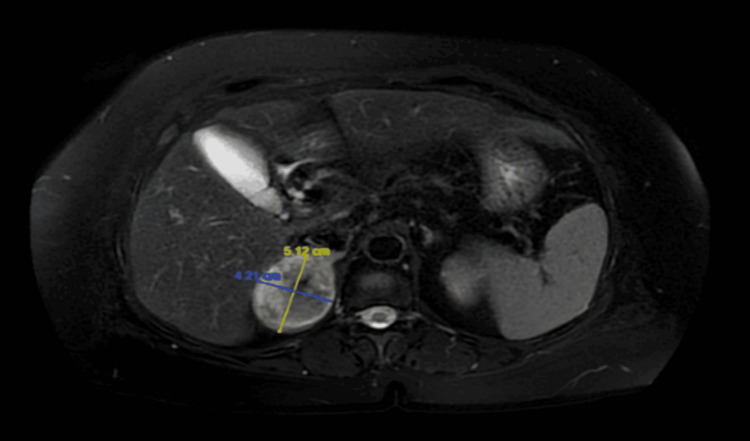
Transverse section of MRI abdomen showing the maximum dimension of the right adrenal mass (T1-weighted)

**Figure 2:**
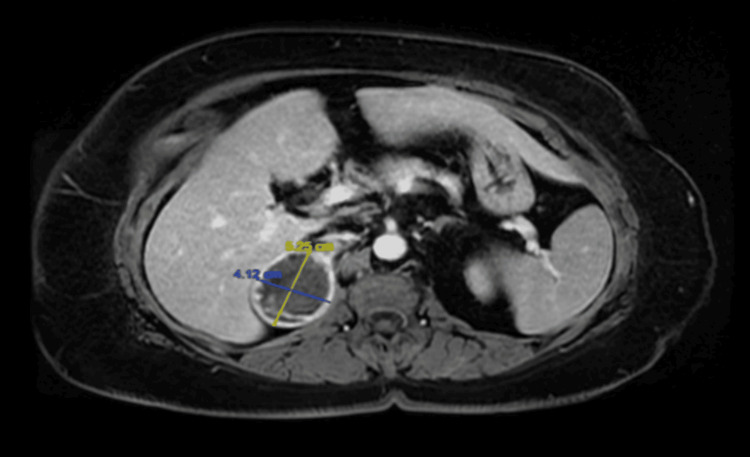
Transverse section of MRI abdomen showing the maximum dimension of the right adrenal mass (T2-weighted)

**Figure 3:**
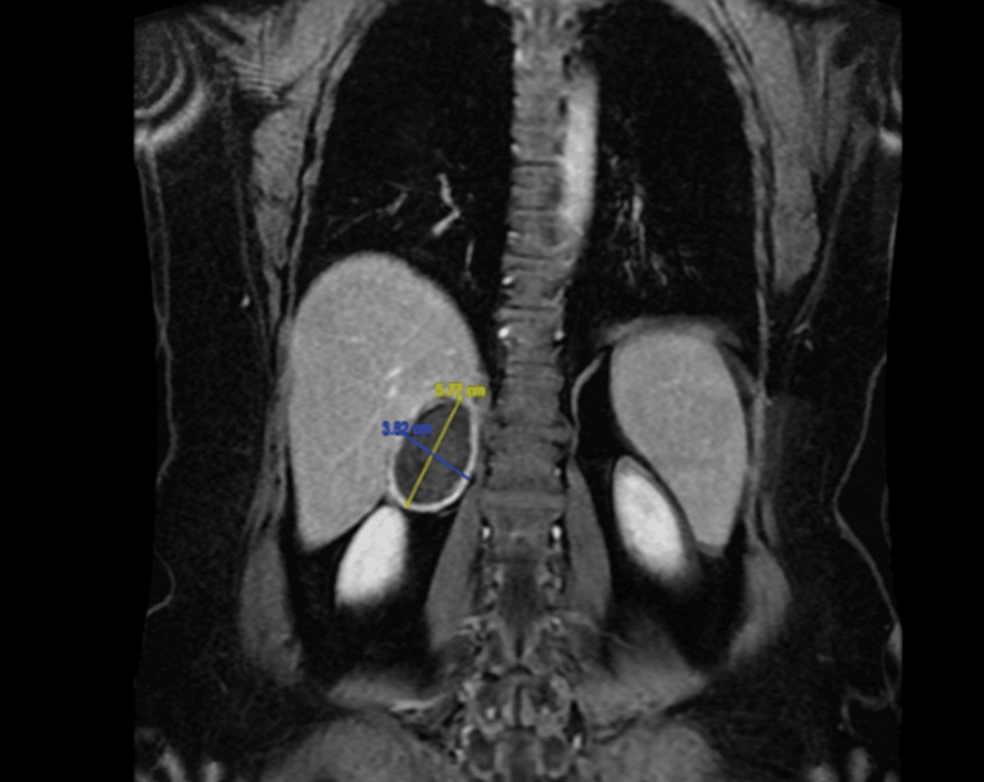
Coronal section of MRI abdomen showing the maximum dimension of the right adrenal mass (T2-weighted)

The workup included radiological studies (magnetic resonance imaging of abdomen and pelvis), which showed a heterogenous enhancing right adrenal mass with a maximum dimension of 5.7 cm (Figures 1-3) The biochemical workup revealed it as a non-functional mass (Table 1).

**Table 1:** Hormonal serum level, which concludes non-functional mass

Hormone	Results	Normal Range
Noradrenaline	337	70-1700 pg/ml
Metanephrine	21	12-60 pg/ml
Cortisol	7.6	5-25 mcg/dl

As part of the pre-operative workup, the patient went for a cardiac workup and was cleared as low risk according to the modified Lee score. Anti-platelet medications (clopidogrel) was held before the surgery. On the day of surgery, prophylactic anticoagulation (heparin 5000 IU) was given subcutaneously two hours before the surgery, and prophylactic antibiotics (2 grams of cefazolin, which is a first-generation cephalosporin) were given upon induction of general anesthesia. The patient was placed in a supine position on the operating table with a sandbag underneath the right flank, and prepped and draped under usual sterile condition. Laparoscopy of the abdomen commenced with insufflation of the abdomen via a 5 mm vesiport, which was inserted following an optical maneuver. Other ports were then inserted under direct vision. The enlarged right adrenal gland was identified, and dissection lateral to the inferior vena cava (IVC) was initiated till the gland was completely exposed using an energy device (LigaSure™, Medtronic plc, Dublin, Ireland), after the adrenal vein was seen, dissected, and hemostasis secured by clipping it. Lastly, the abdomen was deflated, the skin was sutured, and the patient was extubated. The surgery took around two hours and the patient shifted to recovery and then to the surgical ward as per routine. The postoperative period went uneventful, and the patient was discharged on postoperative day 2 and was free from complaints at the outpatient follow-ups. 

**Figure 4:**
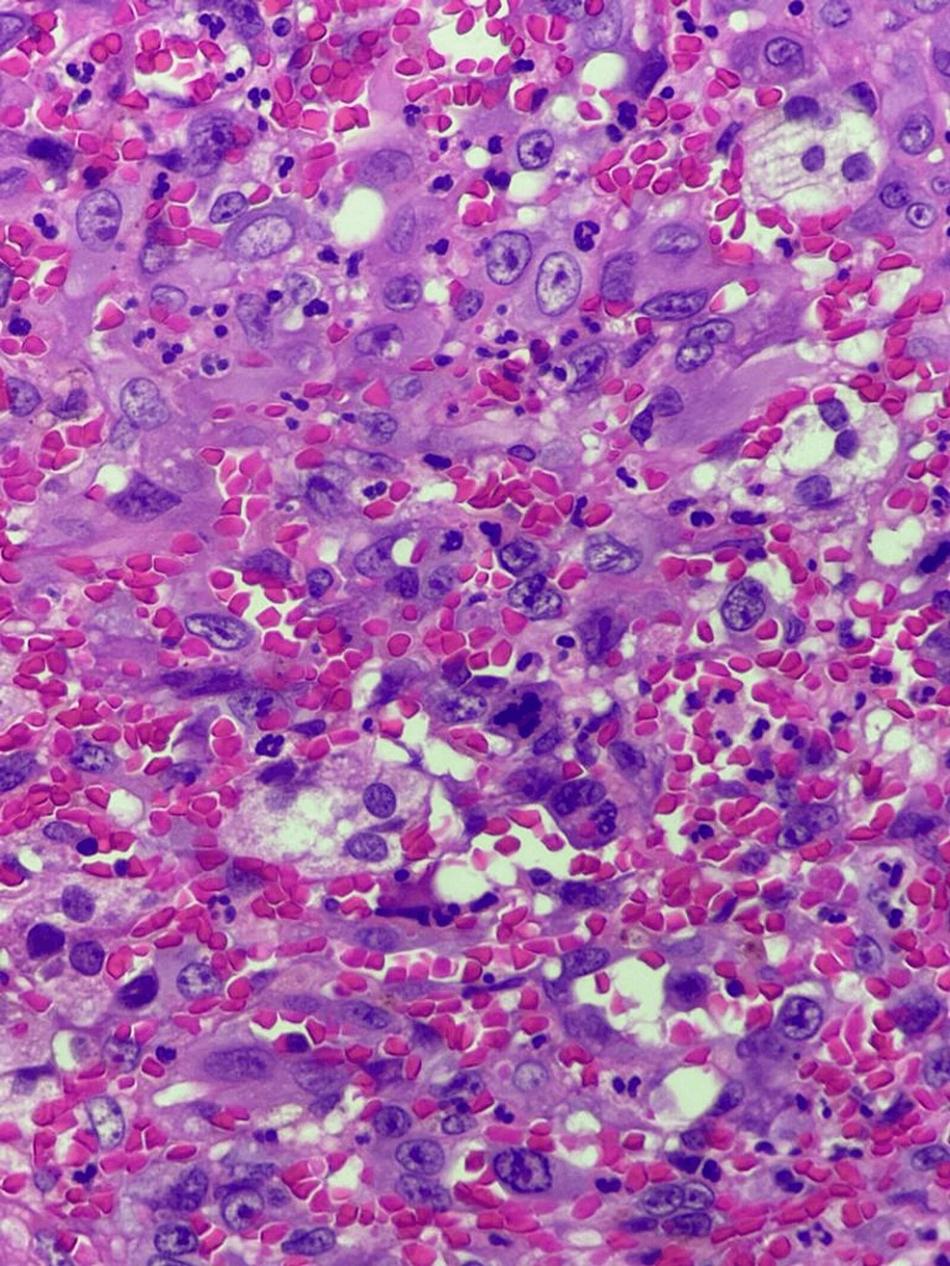
Several atypical mitosis and central necrosis

**Figure 5:**
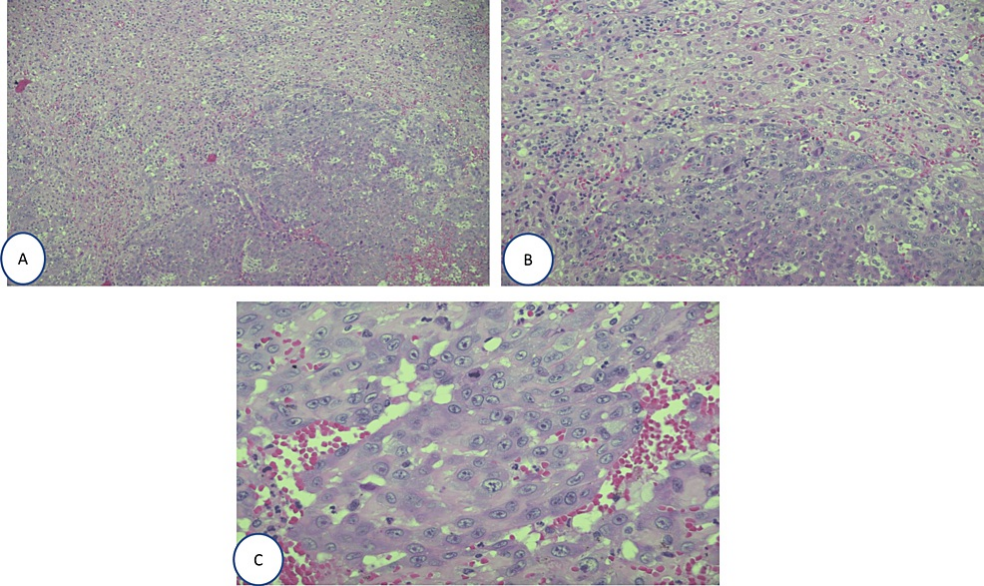
Light microscopy photographs of the tumor showing atypical epithelioid cells. Hematoxylin and eosin stain; (A) x100; (B) x200, and (C) x400 magnification

**Figure 6:**
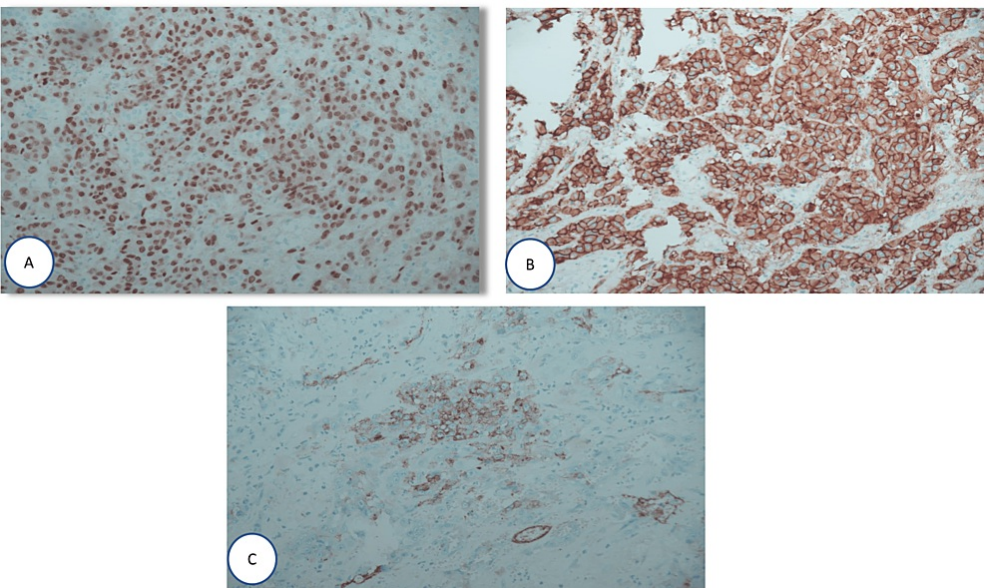
Light microscopy photographs of the tumor showing (A) nuclear positivity to ERG, (B) cell membrane positivity to CD31, and (C) cell membrane positivity to CD34 (x200 immunohistochemistry stain) ERG: electroretinogram

The histological examination of the mass showed irregularly shaped anastomosing vascular channels lined by atypical endothelial cells. These tumor cells are pleomorphic and mitotically active with central necrosis (Figure 4). The immunohistochemistry stains were positive for CD31, CD34, and ERG (Figures 5, 6). After the histopathology report came, our plan was to rule out any metastasis that could change our management. So our workup included computed tomography of the chest, abdomen, and pelvis in addition to a positron emission tomography (PET) scan, which all came negative for metastasis. Therefore, our final diagnosis was primary adrenal angiosarcoma, and the patient was referred to the oncology team for follow-up and further workup accordingly. 

## Discussion

As already mentioned, angiosarcoma is a primary mesenchymal neoplasm of endothelial cell origin with anastomosing vascular channels, and the real reason and trigger behind the formation of angiosarcoma still not known [1]. However, few factors have been found to have a correlation with angiosarcoma like chronic lymphedema, history of radiation therapy, familial angiodysplasia, prior anabolic steroid therapy, and exposure to arsenic, vinyl chloride, or thorotrast [2].

Presentations of the patients with this disease have wide variation and could be completely asymptomatic to nonspecific complaints such as abdominal pain, weight loss, anorexia, and fevers [6]. Many other adrenal diseases could have similar vague presentation like adrenal cortical carcinoma, pheochromocytoma, metastatic carcinoma, and metastatic malignant melanoma [7]. Therefore, diagnosing such a case need full advanced workup including blood tests to assess the functionality of the tumor, radiological investigations, and finally the histopathological confirmation. Radiological investigations are considered the cornerstone for the workup, as it can guide us accordingly regarding the need of chemotherapy or radiotherapy [7]. Also, diagnosing a patient by depending only on histopathology reports is very difficult because the expression of cytokeratin could cause physicians to misdiagnose metastatic carcinoma of the adrenal gland. Using immunohistochemical staining for vascular markers like CD34, FLI1, CD31, factor VIII, and CD34 is highly valuable aid for the diagnosis [8]. 

Moreover, angiosarcoma is considered a high aggressive tumor, and its long-term survival showed poor prognosis with five-year survival rates ranging from 24% to 31% [2,4,9]. Regarding the management and the role of surgical resection, which is considered till now the treatment of choice, even though many factors need to be taken into account before proceeding to it [10,11,12], such as the size of the tumor, which showed only 2% of tumors <4 cm are found to be carcinosis while 6% of tumors < 6 cm and 25% of tumors >6 cm [13]. Regarding choosing the type of surgery, open surgery is still the standard for all patients with resectable tumor and it is the chosen one for any tumors exceeding 10 cm. However, over the last 20 years, the laparoscopic approach has become the preferred choice to the adrenal tumor [13]. All recent studies showed no difference in outcomes when comparing open to laparoscopic approaches, but only when the latter is performed by a trained laparoscopic oncology surgeon who has good experience in adrenal tumors [2,13].

## Conclusions

Angiosarcoma is a very rare and challenging malignant tumor and, thus, our case report illustrates the need for a multidisciplinary team to manage such cases. This team should include medical oncologists, surgical oncologists, radiologists, and pathologists to optimally manage it. Currently, surgical resection is the treatment of choice, with laparoscopy being the preferred form of surgery. 
